# Medium Perfusion Flow Improves Osteogenic Commitment of Human Stromal Cells

**DOI:** 10.1155/2019/1304194

**Published:** 2019-05-02

**Authors:** Alice Pasini, Joseph Lovecchio, Giulia Ferretti, Emanuele Giordano

**Affiliations:** ^1^Laboratory of Cellular and Molecular Engineering “S. Cavalcanti,” Department of Electrical, Electronic, and Information Engineering “G. Marconi” (DEI), Alma Mater Studiorum-University of Bologna, Cesena, Italy; ^2^Advanced Research Center on Electronic Systems (ARCES), Alma Mater Studiorum-University of Bologna, Bologna, Italy; ^3^BioEngLab, Health Science and Technology, Interdepartmental Center for Industrial Research (HST-CIRI), Alma Mater Studiorum-University of Bologna, Ozzano Emilia, Italy

## Abstract

Dynamic culture protocols have recently emerged as part of (bone) tissue engineering strategies due to their ability to represent a more physiological cell environment in vitro. Here, we described how a perfusion flow induced by a simple bioreactor system improves proliferation and osteogenic commitment of human bone marrow stromal cells. L88/5 cells were cultured in poly(methyl methacrylate) custom-milled communicating well plates, in the presence of an osteogenic cocktail containing 1*α*,25-dihydroxyvitamin D3, L-ascorbic acid 2-phosphate, and *β*-glycerophosphate. The dynamic cell culture was maintained under perfusion flow stimulation at 1 mL/min for up to 4 days and compared with a static control condition. A cell viability assay showed that the proliferation associated with the dynamic cell culture was 20% higher vs. the static condition. A significantly higher upregulation of the osteogenic markers runt-related transcription factor 2 (RUNX2), collagen type I (COL1A1), osteocalcin (BGLAP), alkaline phosphatase (ALPL), and osteopontin (SPP1) was detected when the perfusion flow stimulation was administered to the cells treated with the osteogenic cocktail. An in silico analysis showed that in the dynamic cell culture condition (i) the shear stress in the proximity of the cell layer approximates 10^−3^ Pa, (ii) the nutrient and the waste product concentration is more homogeneously distributed than in the static counterpart, and (iii) perfusion flow was associated with higher nutrient consumption. In summary, increased cell proliferation and enhanced early phenotype commitment indicate that dynamic cell culture conditions, delivered via bioreactor systems, produce an enhanced in vitro environment for both basic and translational research in tissue engineering and regenerative medicine.

## 1. Introduction

Obtaining more physiological stem cell (SC) culture conditions to support in vitro cell expansion and/or to prompt a phenotype commitment of interest is a valuable objective for both basic and translational research. In this respect, the ability to provide cell cultures with adequate nutrient supply and waste removal and to administer specific physical cues as differentiating signals would foster the study of molecular SC physiology and be relevant for the design of efficient protocols in tissue engineering (TE) and regenerative medicine (RM). Indeed, the main drawback in traditional static cell cultures is the lack of the sustained native diffusive exchanges. This constraint might both affect the cell availability of glucose, an important nutrient for the generation of cellular energy, and consistently determine the accumulation of toxic metabolites such as lactate that could inhibit cell growth [1]. In this respect, including a perfusion flow would provide to the cells a more physiological environment [2, 3]. Moreover, besides increasing cell seeding and viability, fluid perfusion-induced shear stresses were demonstrated to also drive osteogenic phenotype commitment [4–7], which is otherwise traditionally obtained via supplementation to the culture medium of osteogenic soluble factors, such as ascorbic acid, dexamethasone, and *β*-glicerophosphate [8]. Therefore, the aim of this study is to propose a dynamic cell culture approach via an elementary custom perfusion device, hereafter referred to as a bioreactor system, and evaluate its effects on proliferation and early osteogenic commitment of a human bone marrow stromal cell line. Indeed, an in vitro enhancement of the proliferation and differentiation of SC towards the osteogenic phenotype would result in a more efficient platform for scientific research, while accelerating the biological process of osteogenesis would boost the cell therapy paradigm. The in vitro analysis will be complemented by an in silico study of dynamic culture-imposed conditions to explore if diffusive exchanges and fluid-dependent shear stress values are consistent with the observed biological outcomes.

## 2. Materials and Methods

### 2.1. Bioreactor System Architecture

**Figure 1:**
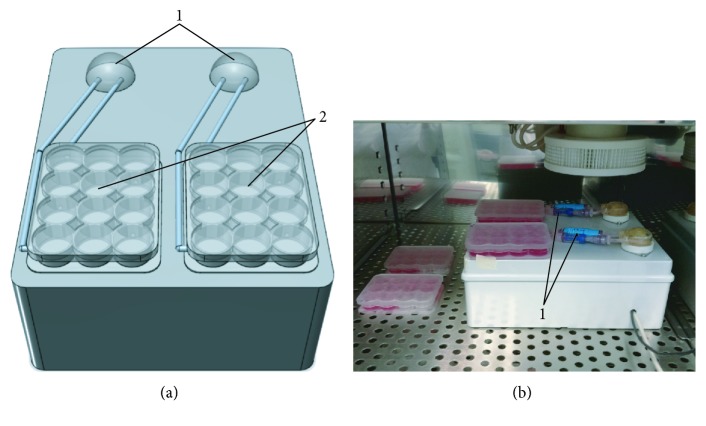
Bioreactor system layout. (a) Front view sketch of the bioreactor system: “1,” peristaltic pumps and “2,” custom PMMA culture plates. (b) Actual prototype: “1,” Spiros®/MicroClave® connectors.

A bioreactor system was designed and built to generate perfusion flow to sustain cell culture. To this aim, two autoclavable WELCO WPM2-S1EACP (WELCO Co. Ltd., Tokyo, Japan) peristaltic pumps, sketched in Figure 1(a)-1, were implemented to promote constant medium delivery to/removal from the cell culture with a perfusion flow rate tunable from a range of 1 to 5 mL/min.

**Figure 2:**
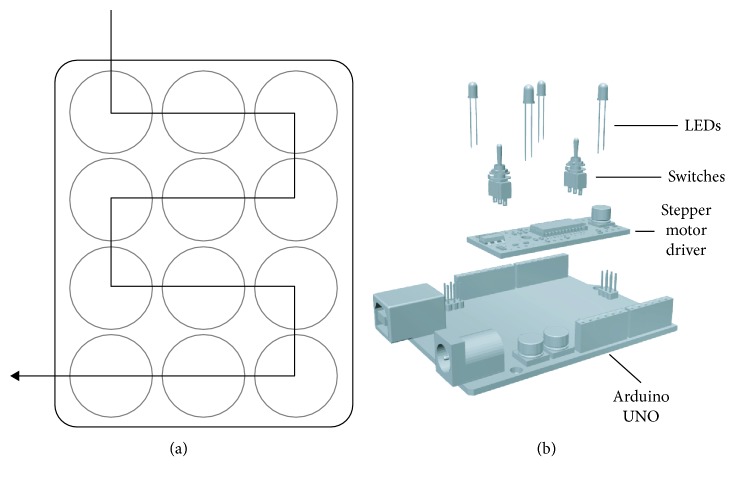
(a) Schematic representation of medium flow direction through the custom PMMA culture plate. (b) Schematic representation of the components of the control unit.

Custom culture chambers, reported in Figure 1(a)-2, were milled in poly(methyl methacrylate) (PMMA, Altuglas® CN 100 10000, Altuglas International, La Garenne-Colombes Cedex, France)—a cheap, easily malleable material, whose biocompatibility for biomedical applications has already been shown [9]—following the geometry of a standard 12-well plate (h12 mm, ⌀22 mm). Holes (⌀2 mm) were drilled between adjacent wells, according to the scheme reported in Figure 2(a), to allow medium to flow through. Two PMMA custom-milled communicating well plates (Figure 1(a)-2) can operate independently to allow comparison of control and osteogenic media supply. Each plate hosts ten glass coverslips maintained under a continuous medium flow.

Each chamber works as an independent detachable unit integrated into a perfusion line via Spiros®/MicroCLAVE® (ICU Medical Inc., San Clemente, CA, USA) connectors, useful to avoid medium leakage and contamination risk during its disconnection from the device for specific evaluations, such as cell observation under the light microscope. Disposable/biocompatible tubes (i.e., Tygon® silicon tubing) are used as perfusion line.

A simple control unit (Figure 2(b)) was implemented in order to manage the device. An EasyDriver (SparkFun Electronics, Boulder, CO, USA) stepper motor driver operates as a guide of the two peristaltic pumps. Two DPDT Mini Power Switches (Lynxmotion Inc., Mirabel, QC, Canada) and four colored LEDs (SparkFun Electronics) are used to allow “play (green),” “pause (red),” “filling (yellow),” or “perfusion (blue)” function surveillance. An Arduino UNO was programmed to control all the components of the device. In this study, a perfusion flow rate of 1 mL/min was administered, but this parameter can be easily adapted from a range of 1 to 5 mL/min, by programming the Arduino UNO at the beginning or during the experiments.

In its final layout, the prototypal bioreactor system shown in Figure 1(b) displays a unibody case (l19, w24, and h9 cm). The device is designed to operate inside a standard cell culture incubator that will maintain physiological pH and temperature.

### 2.2. Cell Culture and Osteogenic Induction

The human bone marrow stromal L88/5 cell line (a kind gift of Prof. Jeanette Maier) was cultured in RPMI 1640 (Euroclone S.p.A., Pero, MI, Italy) supplemented with 10% fetal bovine serum, 2 mM L-glutamine, 100 U/mL penicillin, and 100 mg/mL streptomycin, at 37°C in a humidified 5% CO_2_ incubator. Cells were seeded on standard polystyrene plates or on glass coverslips (⌀18 mm) at a density of 10^5^ cells/cm^2^ and allowed to grow for 48 h to reach confluency. Glass coverslips with confluent cells were then carefully transferred via the use of tweezers to the multiwell PMMA custom-milled plates and cultured in dynamic and static conditions. Cells were stimulated with vehicle or osteogenic differentiation cocktail containing 20 nM 1*α*,25-dihydroxyvitamin D3 (D1530, Sigma-Aldrich, Milano, Italy), 50 *μ*M L-ascorbic acid 2-phosphate (49752, Sigma-Aldrich), and 10 mM *β*-glycerophosphate (50020, Sigma-Aldrich), with media replaced every 48 h. At indicated time points, cells were then processed for biological analysis.

### 2.3. Cell Viability Assay

To test their proliferation, L88/5 cells were seeded on glass coverslips (⌀10 mm) at a density of 5000, 10,000, and 20,000 cells/cm^2^ and allowed to adhere overnight. The day after, the coverslips were transferred to (i) PMMA custom-milled communicating well plates, (ii) PMMA custom-milled noncommunicating well plates, and (iii) standard polystyrene well plates. After 48 and 72 h of cell culture, cell viability was assessed using PrestoBlue® Cell Viability Reagent (A13261 Invitrogen, Thermo Fisher Scientific, Waltham, Massachusetts, USA) following the manufacturer's instructions and using a standard curve to compare experiments. Data are reported as mean value±SEM of six coverslips from two independent biological replicates.

### 2.4. Gene Expression Analysis

Quantitative real-time PCR (qPCR) was used to analyze the mRNA expression level of the osteogenic marker genes osteocalcin (BGLAP), alkaline phosphatase (ALPL), osteopontin (SPP1), runt-related transcription factor 2 (RUNX2), collagen type I (COL1A1), and osteonectin (SPARC). After 2, 4, and 10 days of stimulation with the osteogenic cocktail, total RNA was extracted using the NucleoSpin® RNA (Macherey-Nagel GmbH & Co. KG, Düren, Germany) following the manufacturer's instructions. 500 ng of total mRNA was reverse transcribed to cDNA with the iScript™ cDNA Synthesis Kit (170-8891, Bio-Rad, Segrate, MI, Italy). 5 *μ*L of the 1 : 10-diluted cDNA was then amplified by qPCR using the SsoAdvanced™ SYBR® Green Supermix (172-5261, Bio-Rad). KiCqStart® SYBR® Green Primers (Sigma-Aldrich) were used (H_B2M_1, H_GAPDH_1, H_RUNX2_1, H_COL1A1_2, H_BGLAP_1, H_SPP1_1, and H_SPARC_1). qPCR experiments were performed in duplicate using the CFX Connect™ Real-Time PCR Detection System (Bio-Rad) using a two-step protocol (2 min at 95°C, 40 cycles of 5 s at 95°C, and 30 s at 60°C) followed by a melting step between 95 and 65°C. Data analysis was conducted using the CFX Manager™ Software (Bio-Rad), creating a gene study that uses an interrun calibrator to normalize the variability between the experiments. Data are reported as mean value±SEM of at least three independent biological replicates.

### 2.5. Finite Element Modeling of Nutrient/Waste Diffusion in Dynamic Cell Culture

**Figure 3:**
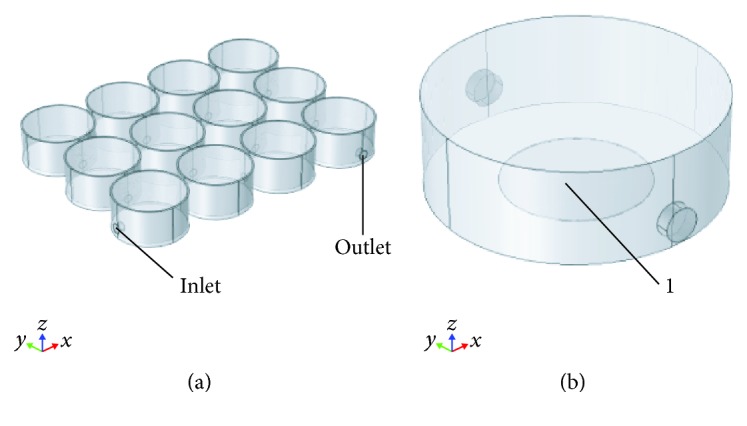
Three-dimensional modeling of the culture unit. (a) PMMA custom-milled communicating well plate. (b) Single well: “1,” glass coverslip.

A Finite Element Model (FEM) (COMSOL Multiphysics® Modeling Software, Stockholm, Sweden) was implemented to investigate fluid velocity profile, shear stress, nutrient consumption, and waste production in static vs. dynamic condition during a 3-day cell culture. The computational model was obtained using primitive geometries and Boolean operations. The whole 12-communicating-well plate (Figure 3(a)) was simulated to study fluid velocity profile. On the other hand, shear stress and nutrient/waste dynamics were calculated using a single well volume (Figure 3(b)). The single well specifications were ⌀22 mm and h7.4 mm; whereas, the single coverslip (Figure 3(b)-1) was ⌀5 mm and h0.1 mm. The inlet/outlet diameter was 1.6 mm.

To approximate the medium properties, the inner volume of the well was modeled as water. Laminar flow, based on Navier-Stokes equations (equation 1), was set up as the physics for the dynamic condition. A velocity of 0.0027 m/s (flow rate 1 mL/min) was used:

(1) $$\begin{array}{c} \left\{\begin{array}{c} \rho \frac{D\vec{u}}{Dt}=-\overset{\to }{\nabla }p+\mu \nabla^{2}\vec{u}+\rho \vec{g},\\ \vec{\nabla }\cdot \vec{u}=0, \end{array}\right. \end{array}$$

where *ρ* is the density, *u* the velocity, *t* the time, *p* the pressure, *μ* the viscosity, and *g* the gravitational acceleration.

Mass transport, based on the second Fick law (equation 2) was modeled for both static and dynamic regimens:

(2) $$\begin{array}{c} \frac{\partial c}{\partial t}=D\;\frac{\partial^{2}c}{\partial x^{2}}, \end{array}$$

where *c* is the concentration, *t* the time, *D* the coefficient diffusion, and *x* the location in the medium.

A sensitivity study of the mesh was performed in order to obtain the most computationally efficient solution. Specific parameters were derived in [10] as reported in Table 1.

Flow rate values of 1, 2, and 5 mL/min were tested aiming to estimate the shear stress values occurring at the bottom of the custom PMMA well and the nutrient consumption.

### 2.6. Statistical Analysis

Statistical analysis was performed using the GraphPad Prism 6 software. Data are presented as mean±SEM of three independent biological replicates, unless otherwise stated. To compare the influences of cell culture conditions over time in a viability assay, two-way ANOVA was used followed by Tukey's multiple comparison test within the same cell seeding density. Student's *t*-test was performed within the same time point, when comparing two data sets during gene expression analysis. To compare the influences of the osteogenic cocktail and mechanical stimulation within the same time point, two-way ANOVA was used followed by Tukey's multiple comparison test. *P* values less than 0.05 were accepted as significant.

## 3. Results and Discussion

Culturing cells within a controlled dynamic environment, where tightly regulated medium perfusion flow sustains cell survival and proliferation, was implemented in this work using an original elementary perfusion bioreactor system. Moreover, a significantly higher and faster upregulation of an osteogenic marker signature was induced when the perfusion flow stimulation was administered to L88/5 cells treated with an accredited osteogenic cocktail.

### 3.1. Effect of Perfusion on Cell Proliferation

**Figure 4:**
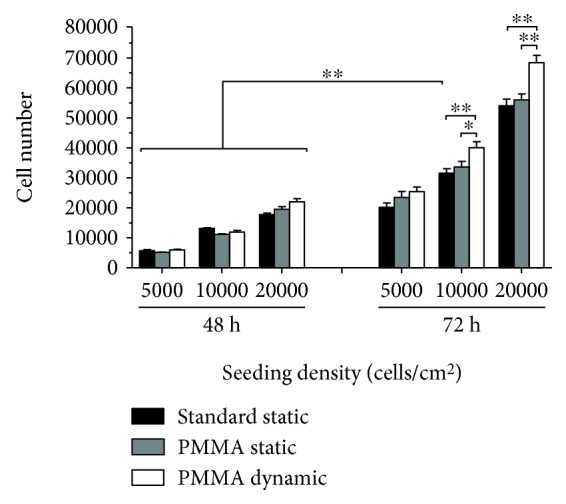
Effect of static and dynamic cell culture conditions over L88/5 cell proliferation. Cell viability of L88/5 cells, seeded at a density of 5000, 10,000, and 20,000 cells/cm^2^ and cultured in standard polystyrene or custom PMMA plates, was evaluated at 48 and 72 h of static or dynamic culture. Data are reported as mean value±SEM of six coverslips from two independent experiments. ^∗^*p* < 0.05 and ^∗∗^*p* < 0.01.

L88/5 cells were seeded overnight on glass coverslips before their transfer into custom UV-irradiated sterile PMMA cell culture plates. Cell viability was evaluated after 48 and 72 h in either static or dynamic culture condition. Three different cell seeding densities (5000, 10,000, and 20,000 cells/cm^2^) were tested. At higher levels (10,000 and 20,000 cells/cm^2^) of cell density, the dynamic cell culture regimen allowed the proliferation of about 20% more cells than the static condition at the later (72 h) time point (Figure 4, grey and white bars, *p* < 0.05), suggesting that in a high-density cell culture, locally reduced nutrient availability and waste product accumulation act as factors limiting cell proliferation [1, 11, 12]. Parallel cultures in standard polystyrene plates (Figure 4, black bars) were carried out as a control condition to rule out any effect of PMMA plates themselves over the basal proliferation capacity of the L88/5 cell line. No difference in cell viability was detected between standard polystyrene and PMMA plates confirming the biocompatibility of the material [9] and of the plate fabrication process.

### 3.2. Osteogenic Differentiation in Standard Cell Culture Condition

**Figure 5:**
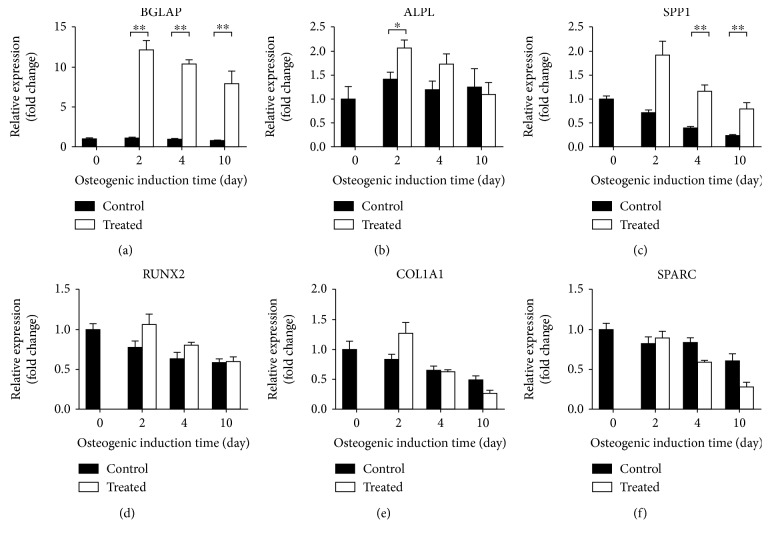
Induction of osteogenic gene expression under standard static cell culture condition conditions. qPCR analysis of osteogenic markers (BGLAP, ALPL, SPP1, RUNX2, COL1A1, and SPARC) was performed on L88/5 cells stimulated with the osteogenic cocktail (20 nM 1*α*,25-dihydroxyvitamin D3, 50 *μ*M L-ascorbic acid 2-phosphate, and 10 mM *β*-glycerophosphate) up to 10 days. B2M and GAPDH were used as reference genes (2^-ΔΔCT^ method). Fold changes from control untreated cells at day 0 were calculated. All data are reported as mean ± SEM of four biological replicates. ^∗^*p* < 0.05 and ^∗∗^*p* < 0.01.

Osteogenic potential of L88/5 cells [13, 14] cultured in standard polystyrene culture plates was verified reproducing the treatment condition published in [15]; thus, upon administration of an osteogenic cocktail (20 nM 1*α*,25-dihydroxyvitamin D3, 50 *μ*M L-ascorbic acid 2-phosphate, and 10 mM *β*-glycerophosphate). Gene expression analysis of osteogenic markers was performed at the 2-, 4-, and 10-day time points and reported in Figure 5. Osteocalcin (BGLAP) expression level was steadily >10-fold compared to its respective control at all the time points tested. A significant 1.5-fold upregulation of alkaline phosphatase (ALPL) gene was scored in cells treated for 2 days, whereas its level of expression became comparable in treated vs. control cells at the later time points (4 and 10 days). On the other hand, the osteopontin (SPP1) mRNA level was significantly higher (about 3-fold) in 4- and 10-day treated cells than in their respective time point control level. The osteogenic markers runt-related transcription factor 2 (RUNX2), collagen type I (COL1A1), and osteonectin (SPARC) were not significantly affected by the treatment. L88/5 cells have been proposed as an easy and inexpensive model of the human bone marrow cell line [13, 14], displaying osteogenic phenotype commitment when appropriately stimulated [15]. The gene expression profile identified in this study highlights that L88/5 cells express basal levels of typical active genes in osteogenic precursors, such as BGLAP, SPP1, or COL1A1, as reported by other groups that also consistently detected the expected lack of expression of collagen types II and III [14]. Our and other published results accredit this cell line and the administered osteogenic cocktail as good experimental models of early osteogenic commitment. The effect induced by the flow perfusion applied via our bioreactor system was thus compared with this static control. Notably, as an additional advantage, L88/5 cells that were originally genetically modified to include the viral DNA sequences of simian virus 40 (SV40) [16] have acquired a short replication time and a clonogenic ability to maintain the same phenotype over passages [13, 14]. These features reduce donor variability and exclude ethical issues associated with the use of primary human MSCs, supporting their use in the validation of standard in vitro protocols and differentiation strategies, such as the perfusion system described in this study.

### 3.3. Effect of Perfusion on Osteogenic Cocktail-Treated Cells

**Figure 6:**
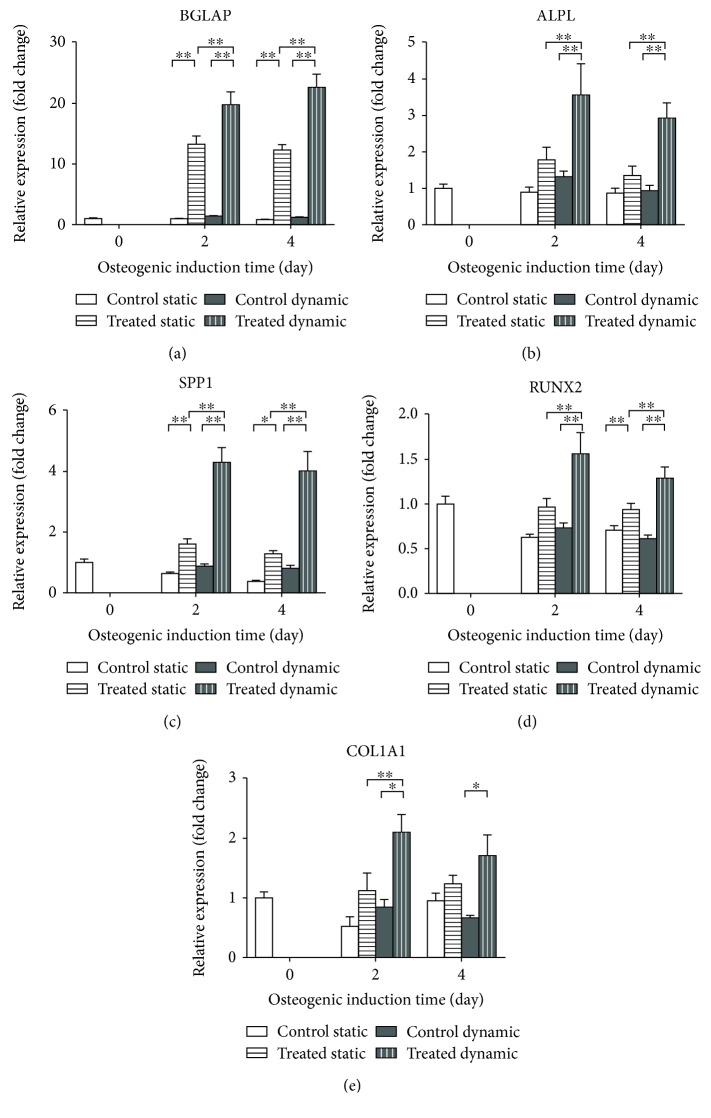
Effect of osteogenic induction on gene expression comparing standard static and dynamic cell culture conditions. qPCR analysis of osteogenic markers (BGLAP, ALPL, SPP1, RUNX2, and COL1A1) was performed on L88/5 cells stimulated with the osteogenic cocktail (20 nM 1*α*,25-dihydroxyvitamin D3, 50 *μ*M L-ascorbic acid 2-phosphate, and 10 mM *β*-glycerophosphate) for 2 and 4 days under static and dynamic cell culture conditions. B2M and GAPDH were used as reference genes (2^-ΔΔCT^ method). Fold changes from control untreated cells at day 0 were calculated. All data are reported as mean±SEM of three biological replicates. ^∗^*p* < 0.05 and ^∗∗^*p* < 0.01.

To investigate the effect of perfusion on osteogenic L88/5 cell potential, gene expression analysis of bone specific markers was performed comparing static vs. dynamic culture conditions. Briefly, cells were grown to confluence on glass coverslips and transferred into custom PMMA plates either without, or with communicating wells to allow media recirculation when perfusion was applied. Cells were then stimulated with the mentioned osteogenic cocktail and collected at day 2 and 4 posttreatment. Gene expression analysis showed that at both the time points studied, all the osteogenic markers were significantly upregulated in the cells cultured in the dynamic condition (Figure 6). In detail, when perfusion was present, both late (BGLAP, ALPL, and SPP1) and early (RUNX2 and COL1A1) osteogenic markers were significantly overexpressed, with respect to the static control condition (about >13-, 3-, 5-, 2-, and 1.5-fold, respectively, *p* < 0.05). The fold-change increase was maintained over the 4 days of induction. In this culture configuration, the standard static condition determines a significant increase of only BGLAP and SPP1 genes 2 days posttreatment (13.6- and 2.5-fold, respectively, compared to control cells; Figure 6). On the other hand, perfusion was also able to determine a significant upregulation of the early markers RUNX2 and COL1A1 already at 2 days of induction whereas prolonged osteogenic induction up to 10 days missed to produce a comparable effect in standard static cell culture, as shown in Figure 5. Moreover, when the perfusion was present, the osteogenic cocktail-induced upregulation of all the osteogenic genes was significantly higher, compared to the static cell culture condition, indicating that the dynamic regimen improves the early osteogenic commitment of this cell line (Figure 6). These results well compare with published evidences in other experimental settings, as reviewed in [17, 18], highlighting how flow perfusion determines a relevant effect in terms of level and speed of phenotype commitment, although variations in cell types, culture conditions, and experimental platforms used increase the complexity of the variables to take into account.

### 3.4. In Silico Evaluation of Perfusion Flow Physics

**Figure 7:**
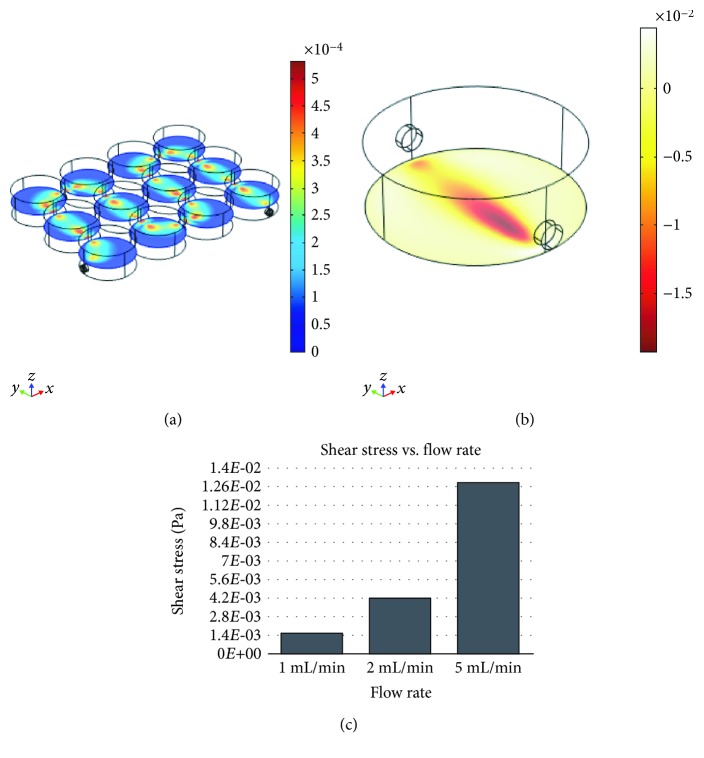
In silico evaluation of the velocity profile in the communicating well plate (a) and of the shear stress in a single well (b) at a flow rate of 1 mL/min. (c) In silico evaluation of the shear stress values corresponding to 1, 2, and 5 mL/min flow rates.

**Figure 8:**
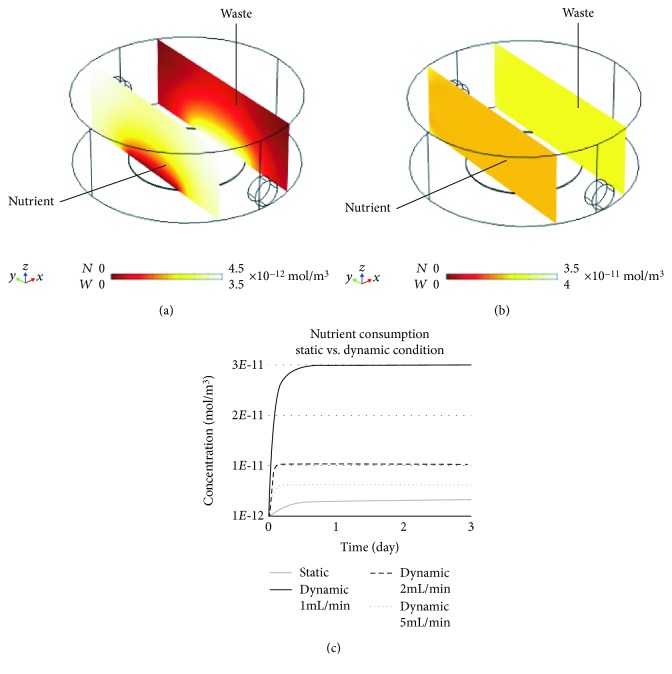
In silico qualitative evaluation of nutrient consumption and waste production distributions within the well volume in static (a) and dynamic (b) conditions; *N*, nutrient consumption; *W*, waste production. (c) Quantitative evaluation of nutrient consumption in static (grey line) vs. dynamic (black line) conditions in the proximity of the cell layer over time (3 days). Δ, time to reach the 95% of the regimen value.

An in silico FEM evaluation was performed to investigate fluid velocity profile (Figure 7(a)), shear stress (Figures 7(b) and 7(c)), nutrient consumption, and waste production (Figure 8). The whole geometry of the PMMA custom-milled communicating well plates was used to evaluate the perfusion velocity profile, while only a single well was considered to evaluate shear stress, nutrient consumption, and waste production, in order to save computational time. Although with an imposed flow rate of 1 mL/min fluid velocity is slightly increased at the well intercommunications, it is maintained within the same magnitude order (10^−4^ m/s) along the whole perfusion path (Figure 7(a)). Flow rate values of 1, 2, and 5 mL/min were tested (Figure 7(c)) aiming to estimate the shear stress value occurring at the bottom of the custom PMMA well. Notably, a flow rate of 5 mL/min entails an average shear stress value of 1.3·10^−2^ Pa. On the other hand, flow rates of 2 mL/min and 1 mL/min entail average shear stress values of 4.2·10^−3^ Pa and 1.6·10^−3^ Pa, respectively; these values are in the range able to induce osteogenic commitment of human MSCs [19, 20].

Figure 8 shows the results of a numerical analysis of nutrient consumption and waste production in static vs. dynamic cell cultures during a 3-day span imposing a flow rate of 1 mL/min. Figures 8(a) and 8(b) illustrate a qualitative evaluation of their spatial distributions within a single well. In the static condition (Figure 8(a)), lower nutrient and higher waste concentration values are present in the proximity of the cell layer, compared to the peripheral area. On the other hand, in the dynamic condition (Figure 8(b)), an homogeneous nutrient/waste concentration distribution is present, suggesting that perfusion enhances diffusive exchanges.  Figure 8(c) shows a quantitative evaluation performed, at different flow rates, to determine the amount of nutrient consumption occurring when static vs. dynamic cell cultures are compared. The time-dependent analysis shows that in dynamic conditions (i) a faster consumption rate is present, as 95% of the steady-state value is reached in 7 h when imposing a flow rate of 1 mL/min, compared with 21 h required for the static condition (Figure 8(c)); (ii) a higher (up to 7.8-fold with respect to static condition) nutrient consumption occurs (Figure 8(c)); and (iii) a lower flow rate (1 mL/min) is associated with higher nutrient consumption, compared with the effects produced by 2 and 5 mL/min flow rates. These results are in line with the finding reported in [21], suggesting that higher flow rates could interfere with diffusive exchanges of nutrient and waste removal. Evidences in the literature show how perfusion bioreactor systems are used to apply fluid flow and to improve mass transport between medium and cells. Perfusion affects cell proliferation and improves osteogenic differentiation of human embryonic stem cell-derived mesenchymal progenitors induced by an osteogenic medium [7]. Fluid shear stress coupled with osteogenic supplements enhances MSC osteogenic differentiation as confirmed by an increased production and improved organization of the extra cellular matrix (ECM) [3]. In summary, the 1 mL/min flow rate used appears as an optimal value in order to obtain a shear stress compatible with osteogenic commitment and higher nutrient consumption.

## 4. Conclusion

Traditional strategies for stem cell (SC) differentiation rely on soluble molecules, such as growth factors and cytokines, that induce a cascade of signalling pathways associated with phenotype commitment. The perfusion system here described shows promising advantages in promoting the early osteogenic commitment of human stromal bone marrow cells. The overexpression of osteogenic markers, indicative of the early phenotype change, is likely to be associated with enhanced diffusive exchanges induced by perfusion, that together with increased waste removal, could improve the transport of nutrients and soluble osteogenic stimuli toward cells, as supported by in silico data. In summary, increased cell proliferation and enhanced early phenotype commitment indicate that dynamic cell culture conditions, delivered via bioreactor systems, produce an enhanced in vitro environment for both basic and translational research in tissue engineering and regenerative medicine. Easy to use, cheap, and versatile devices, such as the one described here, are expected to become a new standard in cell culture, both in research and in clinical laboratories.

## Figures and Tables

**Table 1:** Mass transport parameters.

Parameters	Values	Units
Glucose (initial)	0	mol/m^3^
Waste (initial)	0	mol/m^3^
Nutrient consumption	4.74*E* − 12	mol/s
Waste production	3.4*E* − 12	mol/s
Nutrient diffusion coefficient	3.9*E* − 9	m^2^/s
Waste diffusion coefficient	1.6*E* − 9	m^2^/s

## Data Availability

The data used to support the findings of this study are available from the corresponding author upon request.
